# Morphological characterization and immunohistochemical detection of the proinflammatory cytokines IL-1β, IL-17A, and TNF-α in lung lesions associated with contagious bovine pleuropneumonia

**DOI:** 10.1007/s11250-016-0994-9

**Published:** 2016-02-02

**Authors:** Anja Sterner-Kock, Wolfram Haider, Flavio Sacchini, Anne Liljander, Jochen Meens, Jane Poole, Maria Guschlbauer, Martin Heller, Jan Naessens, Joerg Jores

**Affiliations:** Center for Experimental Medicine, University of Cologne, Medical School, University Hospital of Cologne, Robert-Koch-Straße 10, 50931 Cologne, Germany; Institut für Tierpathologie, Schönhauser Straße 62, 13127 Berlin, Germany; Instituto Zooprofilattico Sperimentale dell’Abruzzo e del Molise “G. Caporale”, Via Campo Boario, 64100 Teramo, Italy; International Livestock Research Institute, Old Naivasha Road, P.O. Box 30709, 00100 Nairobi, Kenya; Institute for Microbiology, Department of Infectious Diseases, University of Veterinary Medicine Hannover, Foundation, 30173 Hannover, Germany; Friedrich-Loeffler-Institut—Federal Research Institute for Animal Health, Naumburger Straße 96a, 07743 Jena, Germany

**Keywords:** CBPP, Contagious bovine pleuropneumonia, Cytokines, Immunocytochemistry, *Mycoplasma mycoides* subsp. *mycoides*, Histopathology

## Abstract

Contagious bovine pleuropneumonia (CBPP), a severe respiratory disease, is characterized by massive inflammation of the lung especially during the acute clinical stage of infection. Tissue samples from cattle, experimentally infected with *Mycoplasma mycoides* subsp. *mycoides* Afadé, were subjected to histopathological and immunohistochemical examination in order to provide insight into innate immune pathways that shape inflammatory host responses. Lung lesions were characterized by vasculitis, necrosis, and increased presence of macrophages and neutrophils, relative to uninfected animals. The presence of three cytokines associated with innate inflammatory immune responses, namely, IL-1β, IL-17A, and TNF-α, were qualitatively investigated in situ. Higher cytokine levels were detected in lung tissue samples from CBPP-affected cattle compared to samples derived from an uninfected control group. We therefore conclude that the cytokines TNF-α and IL-1β, which are prevalent in the acute phase of infections, play a role in the inflammatory response seen in the lung tissue in CBPP. IL-17A gets released by activated macrophages and attracts granulocytes that modulate the acute phase of the CBPP lesions.

**Electronic supplementary material:**

The online version of this article (doi:10.1007/s11250-016-0994-9) contains supplementary material, which is available to authorized users.

## Introduction

Contagious bovine pleuropneumonia (CBPP) is a severe respiratory disease in cattle that is present in many countries in sub-Saharan Africa. CBPP is caused by *Mycoplasma mycoides* subsp. *mycoides* (*Mmm*), a member of the “*Mycoplasma mycoides* cluster” (Fischer et al. 2012) which in addition to *Mmm* comprises four related mycoplasma lineages, i.e., *M. mycoides* subsp. *capri*, *M. capricolum* subsp. *capricolum*, *M. capricolum* subsp. *capripneumoniae*, and *M. leachii*. Besides having a negative impact on the productivity of pastoral cattle farming systems, CBPP is a disease of both regional and international trade, limiting the marketability of cattle in affected regions or countries, consequently resulting in substantial financial losses that negatively impact the livelihood of many people in sub-Saharan Africa (Jores et al. 2013; Thiaucourt et al. 2004b). Cattle infected with *Mmm* can develop acute, subacute, or chronic disease. Acute CBPP is characterized by pyrexia, anorexia, and respiratory signs including rapid and painful breathing and occasionally coughing. Furthermore, large quantities of pleural fluid containing high numbers of mycoplasma are often found during necropsy (Weldearegay et al. 2015). Cattle that exhibit acute disease can either clear the infection, become chronically infected, or die. The current live vaccine against CBPP T1/44 occasionally causes severe side effects at the site of inoculation and, most importantly, lacks efficacy and confers immunity only for up to 1 year, which makes repeated vaccinations essential (Thiaucourt et al. 2004a). The development and subsequent implementation of an improved vaccine, which confers immunity for more than 1.5 years, would greatly benefit a progressive control of CBPP (Ssematimba et al. 2015). However, an increased understanding of the host-pathogen interactions including protective host immune responses is a prerequisite for the rational design of novel vaccines (Jores et al. 2013). Several previous studies have been performed in order to identify how host mechanisms confer immunity to CBPP. The importance of both humoral and T cell-mediated immune responses in mediating protection has been described. Interferon gamma-secreting CD4^+^ T cells have been associated with protection against CBPP during primary infections (Dedieu et al. 2005). These results could however not be confirmed by Jores et al. (2008) and Sacchini et al. (2011), though it is likely that specific CD4^+^ T cell subsets are involved in immunity (Totte et al. 2010, 2008).

Typical CBPP lesions show a fibrinous pleuropneumonia and represent a lobar and lobular pneumonia which usually undergoes acute progression. Classically, the CBPP has four stages. (1) Congestion occurs in the first 24 h postinfection. This stage is characterized histologically by vascular engorgement, intraalveolar fluid, small numbers of neutrophils, and infectious agents. Grossly, the lung is severely hyperemic. (2) Red hepatization or consolidation includes vascular congestion with extravasation of red cells into alveolar spaces, along with increased numbers of neutrophils and fibrin. The filling of alveoli by the exudate leads to a gross appearance of solidification, or consolidation, of the alveolar parenchyma. (3) The stage of grey hepatization is characterized by disintegration of red blood cells, with persistence of the neutrophils and fibrin. The alveoli still appear consolidated, but grossly the color is paler and the cut surface is drier. (4) In the stage of resolution, the pulmonary tissue shows complete recovery. Stages 1 and 2 (congestion, red hepatization) represent more acute pathological findings, whereas stage 3 (grey hepatization) describes chronical pathological findings. Congestion and red and grey hepatization can occur within one individual in parallel at the same time.

Sequestra in the pulmonary parenchyma are a characteristic of chronically diseased animals. They consist of a layer of fibrous tissue enclosing necrotic cells, which is composed of a purulent exudate and live *Mycoplasma* (Caswell and Williams 2007; Schieck et al. 2014). An increased presence of myeloid cells in affected lung tissue was observed in a previous study, though numbers were low (Jores et al. 2008).

Despite a large number of experimental infections performed in the past, documentation of pathological lesions with respect to the in situ presence of distinct host immune cells as well as the presence of cytokines is still lacking. Here, we investigated lungs, mediastinal lymph nodes, and kidneys from ten cattle experimentally infected with CBPP (Sacchini et al. 2011) using standard histologic procedures and immunocytochemistry. The presence of *Mmm* was confirmed using rabbit polyclonal antibodies. Furthermore, we investigated the in situ presence of the inflammatory cytokines tumor necrosis factor alpha (TNF-α), interleukin-1 beta (IL-1β), and interleukin-17A (IL-17A) in experimentally infected and non-infected animals. The data revealed an increase in inflammatory cytokine expression in lung tissues in infected animals compared to non-infected animals.

## Materials and methods

### Ethical considerations

The protocols used in this study were designed and performed in strict accordance with the Kenyan legislation for Animal Experimentation and were approved by the Institutional Animal Care and Use Committee (IACUC reference number 2008.08 [bovine experimental infection] and 2008.14 [rabbit polyclonal antibodies]). Since 1993, the International Livestock Research Institute (ILRI) has complied voluntarily with the United Kingdom’s Animals (Scientific Procedures) Act 1986 that contains guidelines and codes of practice for the housing and care of animals used in scientific protocols.

### Sample collection

The cattle population and the experimental procedure used in the present study have been described in detail elsewhere (Sacchini et al. 2011). In brief, Kenyan Boran bullocks (*Bos indicus*), 14 to 16 months of age at sourcing, were kept at ILRI campus for 2.5 months prior to the transfer to the Animal Biosafety Level 2 facility. Upon arrival to ILRI, the animals were dewormed and treated prophylactically against babesiosis and anaplasmosis. The cattle were revaccinated against lumpy skin disease, anthrax, blackleg, and foot and mouth disease. All animals were tested negative for presence of antibodies against CBPP, East Coast fever, and trypanosomiasis. The cattle were infected by intubation using *M. mycoides* subsp. *mycoides* strain Afadé which has been described elsewhere (Fischer et al. 2015). The animals were monitored daily for up to 30 days post infection (dpi) when the cattle were euthanized and subjected to postmortem analysis.

### Necropsy

The necropsy was done according to a standard procedure (Strafuss 1988). Specimens were taken from lung, mediastinal lymph nodes, and kidneys and were subsequently transferred to tubes containing 5 % buffered formalin solution. Tubes were stored at room temperature until further processing.

### Microbiological and serological analysis

Lung samples and carpal joint fluid and pleural fluid specimens were used for culture isolation of *Mmm* while seroconversion was confirmed using complement fixation test (CFT) (CIRAD, France).

### Polyclonal rabbit antibody production

For antigen preparation, *Mmm* strain Afadé was grown in 100 ml pleuropneumonia-like organism (PPLO) broth (BD Difco™, Germany) supplemented with 20 % horse serum (Sigma, Germany). Cells were pelleted using centrifugation at 5000*g* for 20 min and resuspended in 1 ml of PBS. The cell suspension was ruptured using zirconium beads (0.1 mm diameter, Carl Roth, Germany) in a FastPrep® Instrument (Qbiogene, Germany) three times for 40 s at intensity setting 5.0, followed by heat inactivation at 60 °C for 10 min. A rabbit was immunized with Mmm antigen mixed with the same volume of complete Freund’s adjuvant in the first round and the same volume of incomplete Freund’s adjuvant for the booster injection. Heat-inactivated antigen with adjuvant was injected intradermally and subcutaneously (two depots each). Immunizations were given 4 weeks apart. Preimmunization sera were collected 2 weeks before initial immunization and postimmunization sera were collected 4 weeks after the second immunization.

### Standard histological examination and immunocytochemistry

Formalin-buffered tissue samples were embedded in paraffin wax and subsequently cut into 2-μm slices, using a rotary microtome, and stained with hematoxylin and eosin (HE staining) according to standard protocols.

Immunocytochemistry employing three cytokine markers has been carried out on paraffin-embedded lung tissues. Bovine IL-1β, IL-17A, and TNF-α were detected by using biotinylated rabbit anti-bovine IL-1β polyclonal antibody (article no. ab23778, Abcam, UK), biotinylated rabbit anti-bovine IL-17A polyclonal antibody (article no. PBB0277B-050, Kingfisher Biotech, Inc., USA), and biotinylated rabbit anti-bovine TNF-α polyclonal antibodies (article no. AHP852B, AbD Serotec, Germany), respectively. The SuperVision 2 HRP Single Species (DCS, Germany) was used for detection of biotinylated rabbit and mouse antibodies. Tissues were stained with an irrelevant antibody from the same species to check non-specific binding, and no non-specific binding was evident. Immunoreactive signals were reddish-brown.

Infected animals, showing fibrinous (BD97 and BD107) and non-fibrinous bronchopneumonia (BD92, BD95, BD102, BD105, BD106, BD111, BD115, and BD116), and non-infected animals (control) were compared. Cytokine-positive cells were evaluated within ten non-overlapping, high-power fields (×40 final magnification) on a single microscope by one experienced pathologist. Sections were assessed with the operator blind to clinical details. Macrophages and granulocytes were easily recognizable by staining, size, and morphologic characteristics. Fields containing parts of lymphoid aggregates, massive necrosis, or fibrosis were avoided.

## Results

Clinical symptoms of CBPP such as fever and coughing peaked between 12 and 15 dpi. One animal (BD97) showed severe clinical symptoms and had to be euthanized before the end of the experiment for animal welfare reasons. Infection was confirmed by seroconversion via CFT and isolation of *Mmm* from cultured lung tissues or pleural fluids (Sacchini et al. 2011). At necropsy, typical CBPP lesions were observed in nine out of ten animals. Most pathomorphological lesions were unilaterally present in the left lung with a comparable frequency in the apical and in the diaphragmatic lobe (Sacchini et al. 2011). Only two animals had lesions in the right lung or on both sides. Acute fibrinous bronchopneumonia was only visible in animals BD97 and BD107. In addition, animal BD97 contained a large amount of pleural fluid infected with *Mycoplasma*. The other animals showed mainly chronic lesions with fibrosis and sequestrum formation. No significant differences in the presence of γδ^+^ T cells, monocytes, CD8^+^ T cells, neutrophils, B cells, or granulocytes (Additional file 1) between CBPP animals showing fibrinous or non-fibrinous bronchopneumonia were detected.

Macroscopically visible renal infarcts were present in three animals (BD97, BD107, and BD116) (Table 1).

**Table 1:** Histopathological observations in lungs from cattle with CBPP revealed by hematoxylin eosin-stained tissues

Animal no.	Lung		Middle mediastinal lymph nodes	Kidney
	More acute pathological findings (congestion, red hepatization)^a^	More chronic pathological findings (grey hepatization)^a^		
BD92	Necrosis	Interstitial pneumonia, focal fibrosis	Moderate follicular hyperplasia	–
BD95	Dilated and oedematous interlobular septa, necrosis	Fibrosis, chronic pleurisy, hyperplasia of the BALT	Moderate follicular hyperplasia	–
BD97	Fibrinous bronchopneumonia, dilated and oedematous interlobular septa	–	Moderate follicular hyperplasia	–
BD102	Dilated and oedematous interlobular septa	Hyperplasia of the BALT	Mild to moderate follicular hyperplasia	Mild focal interstitial nephritis
BD105	Dilated and oedematous interlobular septa	Interstitial pneumonia, fibrosis, sequestration	Mild follicular hyperplasia	–
BD106	Dilated and oedematous interlobular septa	Interstitial pneumonia	Mild follicular hyperplasia	Mild focal interstitial nephritis
BD107	Fibrinous bronchopneumonia, dilated and oedematous interlobular septa	Sequestration, fibrosis, hyperplasia of the BALT	Moderate follicular hyperplasia	–
BD111	Necrosis	Interstitial pneumonia, fibrosis	Moderate follicular hyperplasia, multinucleated giant cells	Mild focal interstitial nephritis
BD115	Necrosis	Fibrosis	Mild follicular hyperplasia	–
BD116	Dilated and oedematous interlobular septa	Interstitial pneumonia, fibrosis	Moderate follicular hyperplasia	Mild focal interstitial nephritis

^a^Focal areas of acute and chronic findings are present simultaneously within one individual (see “Introduction” section)

**Fig. 1:**
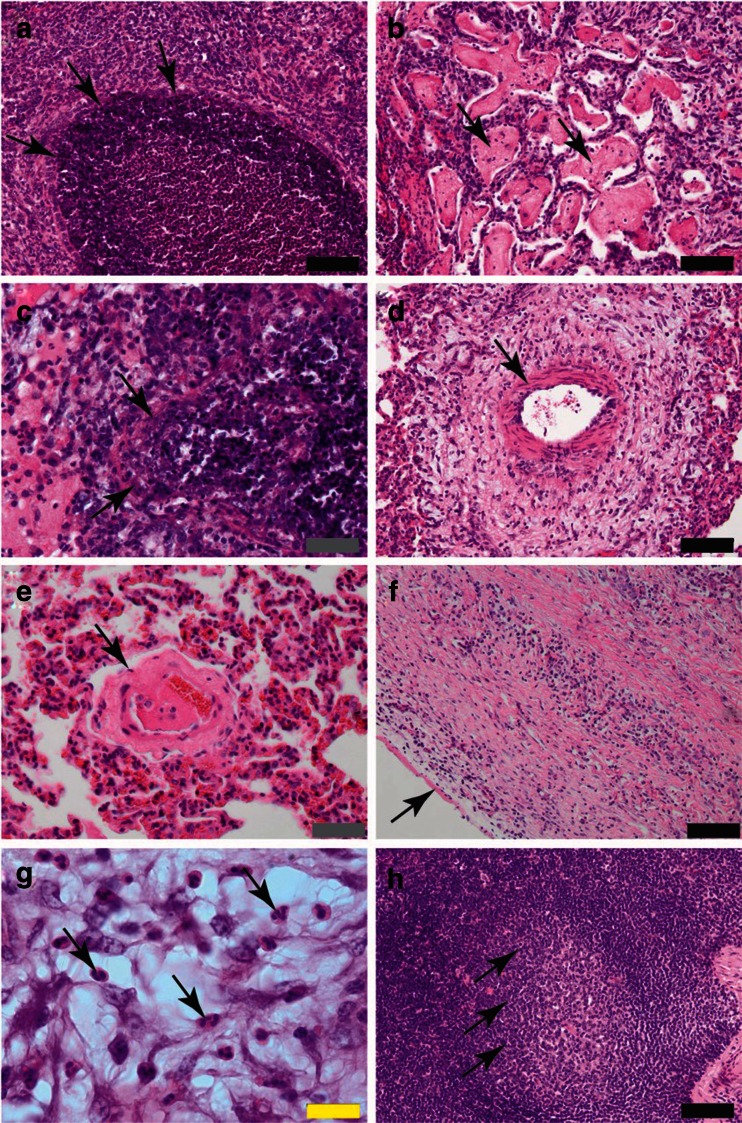
**a** Lung—**a** shows severe, necrotizing bronchiolitis, with severe dense cellular, necrotic debris obstructing the bronchiolar lumen. The remaining bronchiolar wall shows loss of epithelial cells and severe infiltration of pulmonary and interstitial mixed inflammatory infiltrate with loss of pulmonary structure. *Arrows* indicate delineation of bronchiolar lining. **b** Lung—**b** shows intraalveolar proteinaceous, fibrinous precipitates (*arrows*) with loss of pulmonary architecture as well as thickened alveolar septa with mixed inflammatory, interstitial infiltrates within alveolar septa and interstitial space. **c** Lung—c shows a severe, necrotizing vasculitis of a pulmonary arteriole with loss of vascular wall (*arrows* indicate remnants of necrotic vascular wall) and severe, necrotic, cellular debris obliterating the vascular lumen. The perivascular interstitial space shows perivascular, mixed inflammatory cells and protein precipitates. **d** Lung—**d** shows perivascular pulmonary fibrosis extending into the interstitial space, with immature extracellular matrix and mixed inflammatory cells. *Arrow* indicates arterial media hypertrophy. **e** Lung—**e** shows an intrapulmonary arteriole with an adhesive, lumen-obstructing thrombus and media hypertrophy. *Arrow* indicates wall of arteriole. **f** Lung—**f** shows severe chronic, diffuse pleuritis with extensive subpleural fibrosis and neovascularization and loss of pulmonary architecture. *Arrow* indicates pleural surface. **g** Lung—**g** shows an interstitial inflammatory infiltrate composed of neutrophilic and eosinophilic granulocytes (*arrows*) within the interstitial space of the lung. **h** Pulmonary lymphnode—**h** shows a pulmonary lymph node with follicular hyperplasia and activated germinal centers (*arrows*). Size standards are displayed in the lower right corner of each picture: *black* represents 100 μm; *grey* represents 50 μm, and *yellow* represents 20 μm

The histological findings in the lungs were characterized by a juxtaposition of more acute lesions in parallel to more chronic morphological lesions. In the lung, bronchi and bronchioli frequently contained cellular necrotic debris with transmural full-thickness necrosis of bronchial and bronchiolar walls (Fig. 1a). Besides fibrinous bronchopneumonia, alveolitis (Fig. 1b), and fibrinous pleuritis, dilated and oedematous, interlobular septa occurred in these CBPP pulmonary lesions. Severe leukocytoclastic vasculitis with full-thickness necrosis and lysis of the vascular wall was observed within the pulmonary parenchyma (Fig. 1c).

**Fig. 2:**
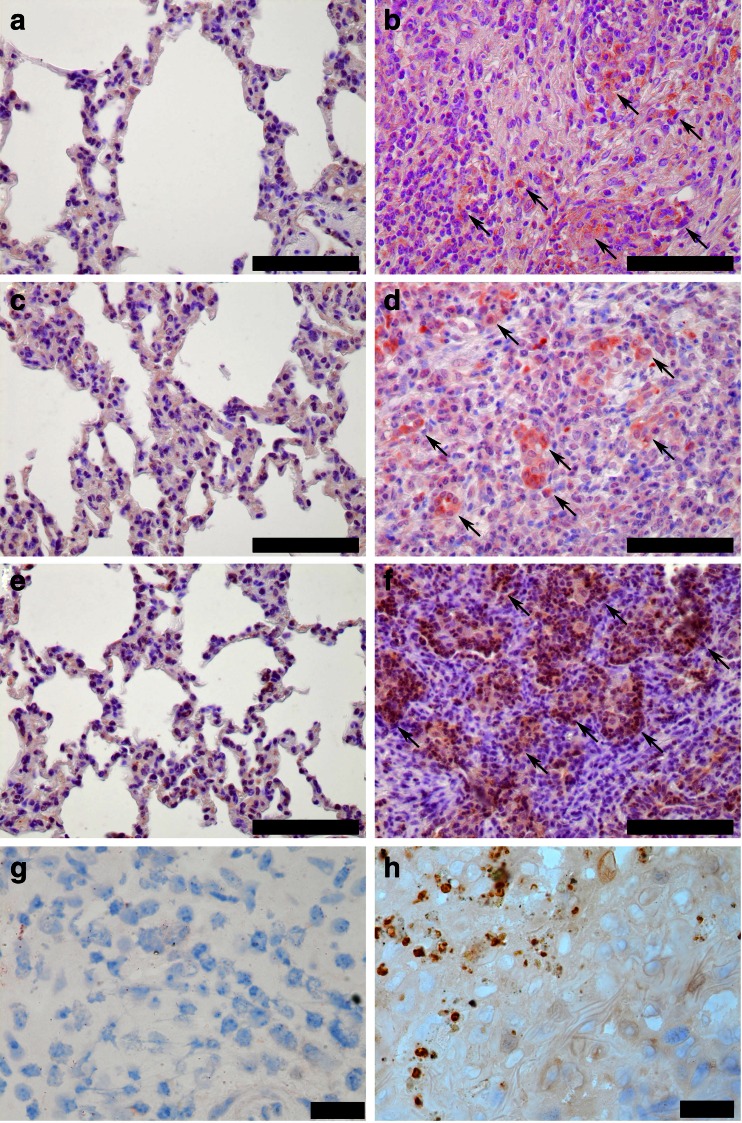
Representative qualitative results of the detection of the cytokines IL-1β, IL-17A, and TNF-α. The following polyclonal antibodies were used. **a Anti- ** IL-1β: Lung: control animal; **b** Anti- IL-1β: Lung: acute CBPP lesion; **c** Anti- IL-17A: Lung: control animal; **d** Anti- IL-17A: Lung: acute CBPP lesion; **e** Anti- TNF-α: Lung: control animal; **f** Anti- TNF-α: Lung: acute CBPP lesion; **g** Anti- *Mycoplasma*: Lung: control animal; **h** Anti- *Mycoplasma*: Lung: acute CBPP lesion. Black bars in the lower right corner of images in **a**–**f** is the size standard representing 100 μm, black bars in images of **g**, **h** represent 20 μm. Arrows indicate positive immunoreactive signals (*reddish-brown*)

Blood vessels in the interstitial septa often showed thrombosis (Fig. 1e) and vasculitis (Fig. 1c). These fibrinous thrombi often contained leukocytes and macrophages. Occasionally progressive organization of connective tissue and neoangiogenesis within immature granulation tissue were evident in the lung (Fig. 1d, f). The stroma contained variable amounts of neutrophils and lymphocytes and occasionally eosinophilic granulocytes (Fig. 1g). Pulmonary lymph nodes of all animals developed a moderate to severe follicular hyperplasia with activated germinal centers (Fig. 1h). Lung tissues were evaluated using three different cytokine-specific markers, and expression of the these three cytokines (TNF-α, IL-1β, and IL-17A) were investigated. At least three different sections of the lung showing pathomorphological changes were evaluated semiquantitatively (Table 2). CBPP lesions were characterized by increased levels of TNF-α, IL-1β, and IL-17A compared to the group of five non-infected animals (Fig. 2, Table 2).

**Table 2:** Semi-quantitative assessment of inflammatory cytokines in the lung of infected and uninfected animals

Animal no.	TNF-α	IL-1β	IL-17A
Negative control group (*n* = 5)	−	−	−
BD92	++	++	++
BD95	++	++	++
BD97	+++	+++	+++
BD102	++	++	++
BD105	++	++	++
BD106	++	++	++
BD107	++	++	++
BD111	++	++	++
BD115	++	++	++
BD116	++	++	++

Immunoreactive signal: ++ moderate (20–40/HPF); +++ severe (≥40/HPF)

## Discussion

This study aimed to characterize morphological lesions in animals experimentally infected with *Mycoplasma mycoides* subsp. *mycoides* in greater detail, by employing HE staining and immunocytochemistry.

The tissue samples investigated were derived from cattle that had been successfully infected as shown by seroconversion and the isolation of the causative agent from all animals (Sacchini et al. 2011). The gross pathological lung lesions reflected the typical pathological patterns described for CBPP (Caswell and Williams 2007). Necrotic areas of the pulmonary lymph nodes have also been previously reported from animals showing acute clinical symptoms of CBPP (Scanziani et al. 1997). Four out of ten animals in the present study showed mild focal interstitial renal inflammation and necrosis (Table 1) as reported elsewhere (Grieco et al. 2001). Seven out of ten infected animals showed mild inflammation of the liver parenchyma and interstitium. Other investigated organs did not show any noteworthy findings. The lesions characterized by HE staining confirmed previous findings related to CBPP. Altogether, the histological patterns observed in the animals used in this study manifested similar lesions to those observed in natural outbreaks.

The presence of *Mycoplasma* antigen in CBPP lesions was also investigated. Rabbit polyclonal sera were used to detect the presence of antigen. As expected, the presence of *Mycoplasma* antigen in macrophages and in lung tissue was confirmed (Fig. 2h).

The results obtained in this study varied within the group of tested animals and could not be correlated with specific clinical and pathomorphological data of individual animals. Thus, use of increased animal numbers in future studies may result in correlations not identified here.

Since it has been indicated that antibody levels can correlate with disease severity (Mulongo et al. 2015; Nicholas et al. 2009; Schieck et al. 2014), immune-mediated vasculitis might be involved in the pathogenesis of CBPP, which will require confirmation in future studies. It has been stated that the presence of vasculitis in CBPP might be caused by antigen-antibody complexes deposited in arterial walls (Thiaucourt et al. 2004b). TNF-α plasma levels are increased in acute diseased animals compared to animals with mild disease symptoms (Sacchini et al. 2012). Therefore, the presence of TNF-α levels in the target tissue was evaluated. As expected, elevated levels of TNF-α in the lung tissues confirmed an involvement of the proinflammatory cytokine in pathogenesis of CBPP as previously proposed (Sacchini et al. 2012). Alveolar macrophages, residing in the lungs, are likely to initiate a response after interaction with the pathogen and be a source of TNF-α and proinflammatory cytokines as indicated in previous in vitro experiments (Jungi et al. 1996). However, it is possible that a component of the CD8^+^ T cell response also contributed to increased TNF-α levels (Hamada et al. 2013). TNF-α is crucial in protection against other respiratory infections such as tuberculosis. It has been shown that TNF-α released from both macrophages and T cells contributes in distinct ways to the sustained control of *Mycobacterium tuberculosis* (Allie et al. 2013). A role for this cytokine in the control of CBPP should be tested in future studies.

The presence of macrophages and granulocytes in CBPP lesions prompted us to investigate the presence of the cytokines IL-1β and IL-17A, which are markers for innate immune responses and granulocyte-attracting molecules, respectively (McAleer and Kolls 2014). Both cytokines were evidently present in affected lung tissue and are likely to mediate the immune responses observed in CBPP. An increase in IL-1β is likely to be induced by alveolar macrophages or dendritic cells after contact with mycoplasma molecules and will stimulate the acute phase response and secretion of IL-17 by γδ^+^ T cells, natural killer cells, or CD4^+^ T cells in case of a memory immune response. One possible hypothesis is that IL-17 has major protective roles in adaptive and innate responses (McAleer and Kolls 2014). In respiratory infections, IL-17 recruits neutrophils to the site of inflammation and affects lung epithelial cells by stimulating antimicrobial peptide secretion and mucus production (Newcomb et al. 2013). In addition, memory Th17 cells are important in adaptive immunity. However, in some cases IL-17 can drive acute lung injury, as in experimental infection with the influenza strain H1N1 (Li et al. 2012). If HIN1 is a representative model, it is therefore possible that in CBPP, IL-17 could contribute to both protective and deleterious effects.

To conclude, innate inflammatory responses have been observed in affected lungs. The main drivers of the innate response are likely to be macrophages, granulocytes, and to a lesser extent epithelial cells. We confirmed elevated in situ levels of the three proinflammatory cytokines IL-1β, IL-17A, and TNF-α in lung tissues from cattle experimentally infected with *Mmm*.

## Electronic Supplementary Material

Supplementary File 1 Comparison of fibrinous and non-fibrinous CBPP lesions with respect to the presence of γδ T-cells, monocytes, granulocytes, CD8^+^ T-cells, neutrophils and B-cells (XLSX 21 kb)

## Abbreviations

BALT: Bronchoalveolar lymphoid tissue
CBPP: Contagious bovine pleuropneumonia
dpi: Days post infection
IACUC: Institutional Animal Care and Use Committee
IL-1β: Interleukin-1 beta
IL-17A: Interleukin-17A
ILRI: International Livestock Research Institute
*Mmm*: *Mycoplasma mycoides* subsp. *mycoides*
TNF-α: Tumor necrosis factor alpha

## References

[CR1] Allie N, Grivennikov SI, Keeton R, Hsu NJ, Bourigault ML, Court N, Fremond C, Yeremeev V, Shebzukhov Y, Ryffel B, Nedospasov SA, Quesniaux VF, Jacobs M (2013). Prominent role for T cell-derived tumour necrosis factor for sustained control of *Mycobacterium tuberculosis* infection. Scientific Reports.

[CR2] Caswell JL, Williams KJ, Maxie MG (2007). Respiratory System. Jubb, Kennedy, and Palmer’s Pathology of domestic animals, 2007.

[CR3] Dedieu L, Balcer-Rodrigues V, Yaya A, Hamadou B, Cisse O, Diallo M, Niang M (2005). Gamma interferon-producing CD4 T-cells correlate with resistance to *Mycoplasma mycoides* subsp. *mycoides* S.C. infection in cattle. Veterinary Immunology and Immunopathology.

[CR4] Fischer, A., Santana-Cruz, I., Hegerman, J., Gourle, H., Schieck, E., Lambert, M., Nadendla, S., Wesonga, H., Miller, R.A., Vashee, S., Weber, J., Meens, J., Frey, J., Jores, J., 2015. High quality draft genomes of the *Mycoplasma mycoides* subsp. *mycoides* challenge strains Afadé and B237, Standarts in Genomic Sciences, 10, 89. 10.1186/s40793-015-0067-0 PMC4625578 26516405

[CR5] Fischer A, Shapiro B, Muriuki C, Heller M, Schnee C, Bongcam-Rudloff E, Vilei EM, Frey J, Jores J (2012). The Origin of the ‘*Mycoplasma mycoides* Cluster’ Coincides with Domestication of Ruminants. PloS ONE.

[CR6] Grieco V, Boldini M, Luini M, Finazzi M, Mandelli G, Scanziani E (2001). Pathological, immunohistochemical and bacteriological findings in kidneys of cattle with contagious bovine pleuropneumonia (CBPP). Journal of Comparative Pathology.

[CR7] Hamada H, Bassity E, Flies A, Strutt TM, Garcia-Hernandez Mde L, McKinstry KK, Zou T, Swain SL, Dutton RW (2013). Multiple redundant effector mechanisms of CD8+ T cells protect against influenza infection. Journal of Immunology.

[CR8] Jores J, Mariner JC, Naessens J (2013). Development of an improved vaccine for contagious bovine pleuropneumonia: an African perspective on challenges and proposed actions. Veterinary Research.

[CR9] Jores J, Nkando I, Sterner-Kock A, Haider W, Poole J, Unger H, Muriuki C, Wesonga H, Taracha EL (2008). Assessment of *in vitro* interferon-gamma responses from peripheral blood mononuclear cells of cattle infected with *Mycoplasma mycoides* ssp. *mycoides* small colony type. Veterinary Immunology and Immunopathology.

[CR10] Jungi TW, Krampe M, Sileghem M, Griot C, Nicolet J (1996). Differential and strain-specific triggering of bovine alveolar macrophage effector functions by mycoplasmas. Microbial Pathogenesis.

[CR11] Li C, Yang P, Sun Y, Li T, Wang C, Wang Z, Zou Z, Yan Y, Wang W, Wang C, Chen Z, Xing L, Tang C, Ju X, Guo F, Deng J, Zhao Y, Yang P, Tang J, Wang H, Zhao Z, Yin Z, Cao B, Wang X, Jiang C (2012). IL-17 response mediates acute lung injury induced by the 2009 pandemic influenza A (H1N1) virus. Cell Research.

[CR12] McAleer JP, Kolls JK (2014). Directing traffic: IL-17 and IL-22 coordinate pulmonary immune defense. Immunological Reviews.

[CR13] Mulongo M, Frey J, Smith K, Schnier C, Wesonga H, Naessens J, McKeever D (2015). Vaccination of cattle with the N terminus of LppQ of *Mycoplasma mycoides* subsp. *mycoides* results in type III immune complex disease upon experimental infection. Infection and Immunity.

[CR14] Newcomb DC, Boswell MG, Sherrill TP, Polosukhin VV, Boyd KL, Goleniewska K, Brody SL, Kolls JK, Adler KB, Peebles RS (2013). IL-17A induces signal transducers and activators of transcription-6-independent airway mucous cell metaplasia. American Journal of Respiratory Cell and Molecular Biology.

[CR15] Nicholas RA, Ayling RD, McAuliffe L (2009). Vaccines for *Mycoplasma* diseases in animals and man. Journal of Comparative Pathology.

[CR16] Sacchini F, Luciani M, Salini R, Scacchia M, Pini A, Lelli R, Naessens J, Poole J, Jores J (2012). Plasma levels of TNF-alpha, IFN-gamma, IL-4 and IL-10 during a course of experimental contagious bovine pleuropneumonia. BMC Veterinary Research.

[CR17] Sacchini F, Naessens J, Awino E, Heller M, Hlinak A, Haider W, Sterner-Kock A, Jores J (2011). A minor role of CD4+ T lymphocytes in the control of a primary infection of cattle with *Mycoplasma mycoides* subsp. *mycoides*. Veterinary Research.

[CR18] Scanziani E, Paltrinieri S, Boldini M, Grieco V, Monaci C, Giusti AM, Mandelli G (1997). Histological and immunohistochemical findings in thoracic lymph nodes of cattle with contagious bovine pleuropneumonia. Journal of Comparative Pathology.

[CR19] Schieck E, Liljander A, Hamsten C, Gicheru N, Scacchia M, Sacchini F, Heller M, Schnee C, Sterner-Kock A, Hlinak A, Naessens J, Poole J, Persson A, Jores J (2014). High antibody titres against predicted *Mycoplasma* surface proteins do not prevent sequestration in infected lung tissue in the course of experimental contagious bovine pleuropneumonia. Veterinary Microbiology.

[CR20] Ssematimba A, Jores J, Mariner JC (2015). Mathematical modelling of the transmission dynamics of contagious bovine pleuropneumonia reveals minimal target profiles for improved vaccines and diagnostic assays. PloS ONE.

[CR21] Strafuss AC (1988). Necropsy: procedures and basic diagnostic methods for practicing veterinarians.

[CR22] Thiaucourt F, Aboubakar Y, Wesonga H, Manso-Silvan L, Blanchard A (2004). Contagious bovine pleuropneumonia vaccines and control strategies: recent data. Developmental Biology (Basel).

[CR23] Thiaucourt F, Van der Lugt JJ, Provost A, Coetzer JAW, Tustin RC (2004). Contagious bovine pleuropneumonia. Infectious diseases of livestock, 2004b.

[CR24] Totte P, Duperray C, Dedieu L (2010). CD62L defines a subset of pathogen-specific bovine CD4 with central memory cell characteristics. Developmental and Comparative Immunology.

[CR25] Totte P, Rodrigues V, Yaya A, Hamadou B, Cisse O, Diallo M, Niang M, Thiaucourt F, Dedieu L (2008). Analysis of cellular responses to *Mycoplasma mycoides* subsp. *mycoides* small colony biotype associated with control of contagious bovine pleuropneumonia. Veterinary Research.

[CR26] Weldearegay, Y.B., Pich, A., Schieck, E., Liljander, A., Gicheru, N., Wesonga, H., Thiaucourt, F., Kiirika, L.M., Valentin-Weigand, P., Jores, J., Meens, J., 2016. Proteomic characterization of pleural effusion, a specific host niche of *Mycoplasma mycoides* subsp. *mycoides* from cattle with contagious bovine pleuropneumonia (CBPP), Journal of Proteomics, 131, 93–103. 10.1016/j.jprot.2015.10.016 26476145

